# How may the basal ganglia contribute to auditory categorization and speech perception?

**DOI:** 10.3389/fnins.2014.00230

**Published:** 2014-08-01

**Authors:** Sung-Joo Lim, Julie A. Fiez, Lori L. Holt

**Affiliations:** ^1^Department of Psychology, Carnegie Mellon UniversityPittsburgh, PA, USA; ^2^Department of Neuroscience, Center for the Neural Basis of Cognition, University of PittsburghPittsburgh, PA, USA; ^3^Department of Neuroscience, Center for Neuroscience, University of PittsburghPittsburgh, PA, USA; ^4^Department of Psychology, University of PittsburghPittsburgh, PA, USA

**Keywords:** speech category learning, perceptual learning, basal ganglia, speech perception, categorization, plasticity

## Abstract

Listeners must accomplish two complementary perceptual feats in extracting a message from speech. They must discriminate linguistically-relevant acoustic variability and generalize across irrelevant variability. Said another way, they must *categorize* speech. Since the mapping of acoustic variability is language-specific, these categories must be learned from experience. Thus, understanding how, in general, the auditory system acquires and represents categories can inform us about the toolbox of mechanisms available to speech perception. This perspective invites consideration of findings from cognitive neuroscience literatures outside of the speech domain as a means of constraining models of speech perception. Although neurobiological models of speech perception have mainly focused on cerebral cortex, research outside the speech domain is consistent with the possibility of significant subcortical contributions in category learning. Here, we review the functional role of one such structure, the basal ganglia. We examine research from animal electrophysiology, human neuroimaging, and behavior to consider characteristics of basal ganglia processing that may be advantageous for speech category learning. We also present emerging evidence for a direct role for basal ganglia in learning auditory categories in a complex, naturalistic task intended to model the incidental manner in which speech categories are acquired. To conclude, we highlight new research questions that arise in incorporating the broader neuroscience research literature in modeling speech perception, and suggest how understanding contributions of the basal ganglia can inform attempts to optimize training protocols for learning non-native speech categories in adulthood.

## Introduction

Speech is a highly variable signal. A central challenge for listeners is discovering how this variability maps to language. A change in pitch may be a linguistically irrelevant deviation arising from emotion, or a telling acoustic cue to whether the sound signaled *beach* or *peach*. This is an example of *categorization*, in that potentially discriminable sounds come to be treated as functionally equivalent classes defined by relevant features (see Holt and Lotto, 2010, for a review). Because this perceptual mapping of sounds is specific to linguistic categories (e.g., consonant and vowel phonemes), one must learn speech categories through experience with the native language. Infants begin to learn native-language speech categories within their first year; exposure to native speech input warps speech perception, enhancing discrimination across native speech categories but diminishing within-category discrimination (Kuhl et al., 1992, 2006), and discrimination of non-native categories not present in the native language (Werker and Tees, 1984). By adulthood, one becomes “neurally committed” to native-language-specific speech categories (see Kuhl, 2004, for a review), which in turn can lead to profound difficulty in learning non-native speech categories as an adult (Best, 1995; Flege, 1995). This pattern indicates that experience with the native language plays a crucial role in shaping how we perceive speech.

However, relatively less is known about *how* speech categories are acquired through experience. One main challenge to our understanding is gaining experimental control over participants' history of linguistic experience. Adult listeners' perception has already been tuned by long-term native speech experience, the extent of which cannot be fully measured by the experimenter. Likewise, it is impossible to determine even young infants' speech experience. Exposure to native-language speech is substantial in the early postnatal months and speech experience begins even prenatally (Mehler et al., 1988; Moon et al., 1993). This lack of experimental control imposes critical limitations on understanding of the role of language experience on speech category acquisition, and impedes development of a mechanistic framework of how speech categories are learned.

A small, but growing, literature has been motivated by the premise that modeling the challenges of speech category learning using nonspeech sounds can reveal principles of general auditory category learning. Understanding these principles reveals characteristics of auditory learning available to support speech category learning. For instance, by using novel nonspeech sound categories, Holt and Lotto (2006) demonstrated that distributional characteristics of sound category input influence listeners' perceptual weighting of multiple acoustic cues for categorization. This finding led Lim and Holt (2011) to test whether increasing variability along a cue that is inefficient in a second language may lead second language learners to rely upon it less in subsequent speech categorization. They found that in Japanese adults learning English, increasing the distributional variance along the native Japanese listeners' preferred (but non-diagnostic for English) acoustic cue led the listeners to rely on this cue less in subsequent English speech categorization. This example demonstrates that learning about general auditory categorization processes can inform our approaches to understanding speech perception and learning.

This general perspective on speech perception invites consideration of findings from the cognitive neuroscience literature outside of the domain of speech and auditory processing. Parallel lines of general learning research suggest that there are multiple learning systems and corresponding neural structures, with an emphasis on the significant contributions of subcortical structures in learning (e.g., Doya, 1999, 2000; Ashby and O'Brien, 2005; Seger and Miller, 2010). Understanding the involvement of subcortical learning systems is especially important to developing full neurobiological models of speech categorization, because current neurobiological and theoretical models of speech processing have focused mainly on the cerebral cortex (McClelland and Elman, 1986; Hickok and Poeppel, 2004; but see Guenther, 1995; Guenther and Ghosh, 2003; Guediche et al., 2014).

In the present review, we focus on the potential of one such subcortical system—the basal ganglia—to play a role in speech categorization. The basal ganglia have been widely implicated in category learning outside the domain of speech processing. Basal ganglia-mediated category learning research, conducted mostly in the domain of visual categorization, has focused on learning mechanisms at the level of category decision-making (i.e., selecting appropriate motor responses associated with category membership). This contrasts to the general approach in speech categorization research, which has focused largely on learning-induced category representations occurring at the sensory level (e.g., Callan et al., 2003; Golestani and Zatorre, 2004; Liebenthal et al., 2005; Desai et al., 2008; Lee et al., 2012). It is important to note that these differing perspectives likely represent attention to different aspects of a larger system. Thus, they are potentially mutually informative, although as of yet they have not been integrated in the service of understanding categorization. Here, we aim to review these different lines of research from the perspective of how they can inform speech categorization.

We begin by reviewing the functional role of the basal ganglia. We examine research from animal electrophysiology, human neuroimaging, and human behavior to identify characteristics of basal ganglia processing that may be advantageous for speech category learning. We then consider the basal ganglia as a system that may play a role in auditory category learning. We focus on characteristics that can potentially contribute to learning of speech categories and training approaches to promote effective non-native speech category acquisition.

## Overview of the basal ganglia and reinforcement learning

**Figure 1:**
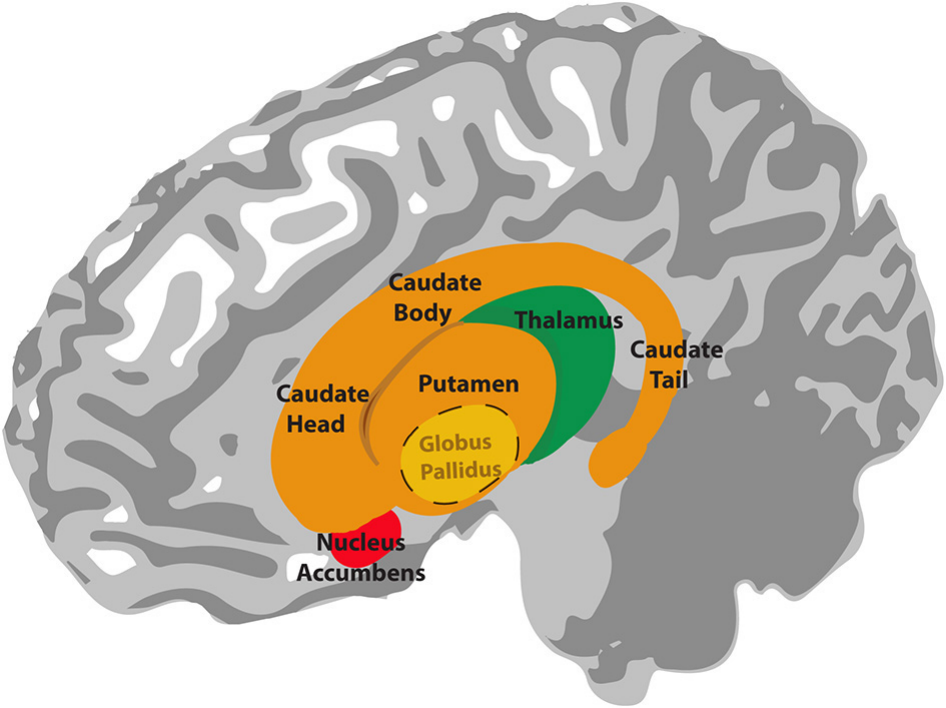
**Illustration of the anatomy of the basal ganglia**. The globus pallidus lies inside the putamen. The thalamus is located underneath the basal ganglia, in the medial position of the brain.

**Figure 2:**
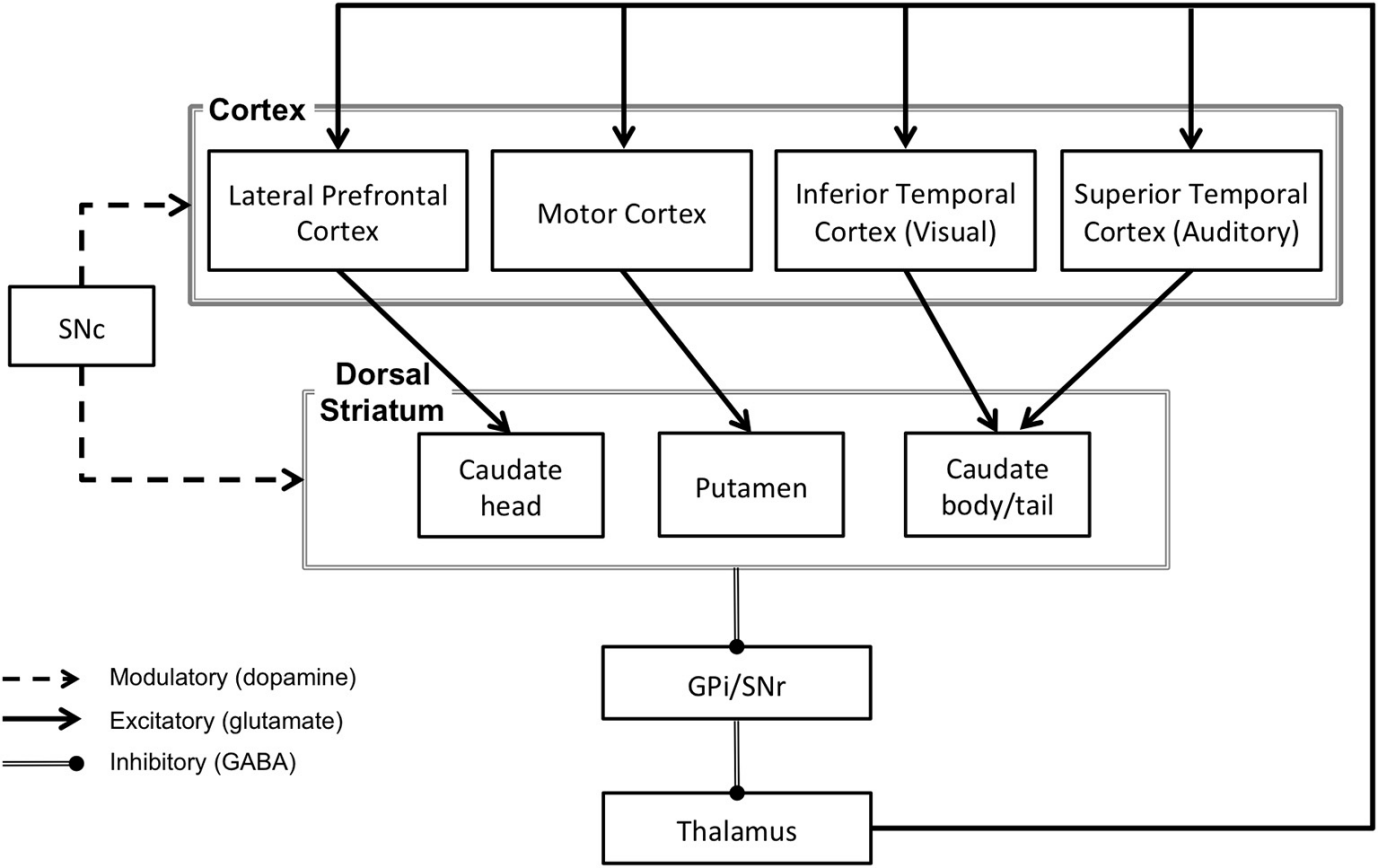
**The direct pathway circuitry of the basal ganglia via the dorsal striatum**. SNc, substantia nigra pars compacta; SNr, substantia nigra pars reticulata; GPi, globus pallidus, internal portion.

The basal ganglia are a collection of subcortical nuclei with a complex circuitry. The input nuclei of the basal ganglia consist of the caudate nucleus and putamen (together referred to as the dorsal striatum) and the nucleus accumbens (considered part of the ventral striatum). The dorsal and ventral striatum receive input from the cerebral cortex and send projections to the output nuclei of the basal ganglia, which include the globus pallidus and the substantia nigra pars reticulata (see Figure 1). The output signals from these nuclei ultimately project back to the cerebral cortex via the thalamus (see Figure 2). This basal ganglia-thalamo-cortical circuitry forms “closed loops,” whereby cortical regions projecting to the basal ganglia receive recurrent feedback projections from the basal ganglia (Alexander et al., 1986) and also “open loops,” whereby cortical regions projecting to the basal ganglia terminate in different cortical regions via the basal ganglia (Joel and Weiner, 1994). In addition to these structures, neurons in the substantia nigra pars compacta and ventral tegmental area play a crucial role in mediating basal ganglia's functions. Dopamingeric projections from these neurons modulate activity of the dorsal and ventral striatum, which ultimately modulate plasticity among the synapses within basal ganglia-thalamo-cortical loops (Reynolds and Wickens, 2002).

The traditional view holds that the basal ganglia are mostly involved in motor-related processing and learning. Basal ganglia circuitry was thought to mainly innervate the primary motor cortex (Kemp and Powell, 1971), which could account for the pronounced movement-related deficits commonly observed among patients with diseases that damage the basal ganglia (e.g., Parkinson's and Huntington's diseases). However, more recent findings have indicated that the basal ganglia nuclei are highly interconnected with widespread areas of the cerebral cortex (Alexander et al., 1986; Middleton and Strick, 2000). This view suggests that the basal ganglia not only influence motor-related processes, but also play an important role in non-motor cognitive functions and a wide range of learning challenges, including perceptual categorization (e.g., Ashby et al., 1998; Hochstenbach et al., 1998; see Lawrence et al., 1998; Saint-Cyr, 2003; Seger, 2008, for reviews).

The basal ganglia are crucially involved in learning appropriate behavioral actions to achieve goals in a given environment. This type of learning can be explained by a computational theory, reinforcement learning, whereby learning emerges as one builds and updates predictions about receiving future rewards. Learning occurs in minimizing the difference between predictions of reward and actual reward, referred as a reward prediction error (Sutton and Barto, 1998). In this way, an unexpected reward or punishment is an indicator that the value of an environmental stimulus (or the best response to it) was not accurately predicted. Therefore, errors in predictions lead to adjustments to predicted value and stimulus-action associations. Based on such predictions, behavior adjusts adaptively to maximize future rewards such that actions leading to rewards are reinforced (i.e., the likelihood of the specific actions increases), whereas incorrect behaviors leading to punishment (or no rewards) are modified. Through this process, reward drives learning of goal-directed actions thereby shaping behavior.

The basal ganglia have been implicated in reinforcement learning by means of the neuromodulatory activity of dopamine neurons located in the midbrain (Schultz et al., 1997; Schultz, 1999; Daw et al., 2005). The dopamine neurons that project to the dorsal striatum are located in the substantia nigra (the pars compacta sector), whereas those that project to the ventral striatum are located in the ventral tegmental area (Nauta et al., 1974; Simon et al., 1979; Swanson, 1982; Amalric and Koob, 1993; Haber and Fudge, 1997). Electrophysiological recording studies on primates by Shultz and colleagues (Schultz et al., 1993, 1997) indicate that dopamine neurons are sensitive to reward prediction. These studies have shown that in the initial phase of learning when rewards are not expected, dopamine neurons fire (i.e., release dopamine) at the onset of reward delivery, but over the course of learning these neurons begin to fire to cues that predict rewarding outcome. When an expected reward is omitted or fails to occur, dopamine levels are depressed (Schultz et al., 1997; Hollerman and Schultz, 1998; Schultz, 1998). A similar pattern of reward-related dopamine neuronal firing is reflected in the activity in the striatum (Hikosaka et al., 1989; Robbins and Everitt, 1992; Schultz et al., 1992, 1993; Tremblay et al., 1998; Schultz, 2000; Berns et al., 2001; McClure et al., 2003).

Computationally, the observed patterns of activity are consistent with the idea that dopamine neurons can signal reward prediction error, which can serve as a teaching signal to drive reinforcement learning. The presumed reward prediction error signals carried by dopamine neurons are thought to modulate the synaptic plasticity of cortico-striatal pathways (Reynolds and Wickens, 2002). Dopamine release can induce long-term potentiation, which effectively strengthens cortico-striatal synapses at the site of release (Wickens et al., 1996; Kerr and Wickens, 2001). This process may be significant in strengthening striatal pathways that encode contexts that predict reward and promote learning of goal-directed actions (i.e., stimulus-response-outcome associations). Therefore, dopamine may be regarded as a learning signal (e.g., Beninger, 1983; Wise and Rompre, 1989; Wickens, 1997; Schultz, 1998, 2002) that reinforces rewarding actions by strengthening stimulus-action associations (Law of Effect, Thorndike, 1911) and mediating relevant cortico-striatal loops to accomplish learning (Houk and Wise, 1995). Conversely, in the case of punishment or omission of expected reward, a relative depression of dopamine levels would induce long-term depression, thus weakening the synapses (Wickens et al., 2003; Calabresi et al., 2007). It is of note that dopamine-mediated learning does not necessarily occur solely through reward prediction error signals processed via the striatum, since dopamine neurons also send direct projections to the cortex (Thierry et al., 1973; Hökfelt et al., 1974, 1977; Lindvall et al., 1974; see Foote and Morrison, 1987, for a review). Nevertheless, the dopaminergic signals through the striatum are likely to be a more robust learning signal, since dopamine neurons disproportionately project to the striatum (Szabo, 1979; Selemon and Goldman-Rakic, 1990; Hedreen and DeLong, 1991; Lynd-Balta and Haber, 1994).

The findings in non-human primates converge with evidence from human neuroimaging studies. Across various learning tasks, including learning non-native phonetic categories (Tricomi et al., 2006), it has been found that activity in the dorsal striatum is modulated according to the valence and the value of feedback that is contingent to one's response actions (i.e., goal-directed behavior) (Elliott et al., 1997, 2004; Koepp et al., 1998; Delgado et al., 2000, 2004; Haruno et al., 2004; O'Doherty et al., 2004; Tricomi et al., 2006). Yet, it is significant to note that rather than responding to response outcomes *per se*, the dorsal striatum exhibits greater activity when individuals perceive the outcomes as contingent on their actions and relevant to their goals (i.e., receiving reward) (Tricomi et al., 2004; Tricomi and Fiez, 2008). Surprisingly, the striatum can even show a reward-like response to negative feedback, if this feedback provides useful information for predicting future rewards (Tricomi and Fiez, 2012). This demonstrates that the striatum is sensitive to the subjective value of information for goal achievements (Tricomi and Fiez, 2008; Han et al., 2010). More generally, these findings suggest that reinforcement learning in humans involves the striatum and it extends into the cognitive domain, as learning can be influenced by high-level thought processes relating to motivation and goal-directed actions.

## Contributions of the basal ganglia to non-native speech category learning

In this section, we consider the challenges involved in learning non-native speech categories and the relative ineffectiveness of passive exposure to non-native speech to improve categorization performance. Then, we review evidence for the effectiveness of directed category training, in which individuals receive goal-relevant feedback about the accuracy of their category judgments. We consider evidence that such training involves an anterior basal ganglia system that drives learning-related changes in non-native speech categorization. Finally, we examine the limitations of directed category training, and consider whether training that encourages the use of procedural learning mechanisms involving a posterior basal ganglia system may be more suited for the perceptual demands of speech category learning.

Adults find it notoriously difficult to learn some non-native speech categories even with extensive training or years of exposure to a foreign language (Gordon et al., 2001; Aoyama et al., 2004; Ingvalson et al., 2011). This difficulty is partly due to interference from expertise with native-language speech categories (Best, 1995; Flege, 1995) developed from long-term experience with their native language since infancy (Werker and Tees, 1984). The case of native Japanese adults' acquisition of English /r/-/l/ has been a prominent example of the difficulty acquiring some non-native speech categories (Goto, 1971; Miyawaki et al., 1975; Werker and Logan, 1985). Whereas English divides the perceptual space into two phonetic categories, /r/ and /l/ as in *rock* and *lock*, there is a single Japanese speech category within a similar perceptual space (Lotto et al., 2004). Having learned this single Japanese category, native Japanese adults have great difficulty distinguishing English /r/-/l/ due to the persistent reliance on the native Japanese perceptual space (Iverson et al., 2003). This difficulty presents important questions regarding the limits and challenges to perceptual plasticity in adulthood.

In attempts to understand adult second language speech category learning, different types of laboratory-controlled training tasks have been used. One common task is unsupervised listening, in which listeners are passively exposed to sound stimuli. Studies using this type of task have shown that listeners' perception is tuned according to the statistical regularity in the input; they become sensitive to the distributional regularities of speech syllables (Maye et al., 2002; Clayards et al., 2008; Goudbeek et al., 2008), correlations between acoustic features defining the units (Idemaru and Holt, 2011), and sequential relationships between syllabic units or tones (Saffran et al., 1996, 1999). However, this type of training fails to facilitate non-native speech category learning in adults. McClelland and colleagues (McClelland et al., 1999; McCandliss et al., 2002; Vallabha and McClelland, 2007) argue that English /r/ and /l/ exemplars are perceptually similar enough to the single Japanese category that hearing English /r/ and /l/ tends to simply activate and strengthen the Japanese category representation among native Japanese adults. They argue that this arises from Hebbian learning principles interacting with the perceptual organization brought about by Japanese language experience. Therefore, unsupervised learning of non-native speech categories may fail unless special steps are taken, such as artificially exaggerating the training stimuli so that they can be perceived as distinct category instances (McCandliss et al., 2002; Tricomi et al., 2006; Ingvalson et al., 2011).

The other dominant, perhaps more effective, training approach to achieve non-native speech category learning is to use directed training that requires overt categorization or identification responses and provides explicit trial-by-trial feedback about the correctness of the response. Directed categorization training has been commonly used to investigate non-native speech category learning (e.g., Logan et al., 1991; Lively et al., 1993, 1994; Bradlow et al., 1997; Wang et al., 1999; Iverson et al., 2005; Francis et al., 2008). Comparisons between passive exposure and directed training tasks have demonstrated an advantage for directed training in learning auditory and speech categories (McCandliss et al., 2002; McClelland et al., 2002; Goudbeek et al., 2008). Although previous training studies have focused on the impact of the acoustic characteristics of training stimuli on learning (Logan et al., 1991; Lively et al., 1993, 1994; Iverson et al., 2005), the learning advantage observed for directed training over passive listening tasks indicates that the details of training are crucial.

Using fMRI, Tricomi et al. (2006) demonstrated that directed category training of non-native speech categories engages the basal ganglia (i.e., the striatum), as compared to a condition without performance feedback. The findings illustrated that the nature of the training task engaged different neural processes and learning systems. Performance feedback may potentially play a crucial role in informing the *functional distinctiveness* of non-native speech categories in traditional laboratory training tasks. Through corrective feedback that encourages distinct action associations (e.g., button presses) for the categories, one's actions are shaped to respond differently to these sound categories, thereby assigning distinct behavioral significance to the sounds.

It is notable that non-native speech category learning in adulthood occurs with directed categorization training, but learning gains are relatively modest even across multiple weeks of extensive training (e.g., Logan et al., 1991; Lively et al., 1993; Bradlow et al., 1997; Iverson et al., 2005). Given the literature reviewed above, which demonstrates that task and stimulus details can be influential in engaging different learning systems, there is the possibility that overt categorization tasks with explicit feedback may fail to tap into the most effective learning mechanisms for adult speech category learning.

One of the main challenges of speech perception and categorization is to map highly variable sound exemplars distributed across multiple acoustic dimensions onto linguistically-relevant phonemic categories (see Holt and Lotto, 2010, for a review). Speech categories are inherently multidimensional such that no single acoustic cue or dimension is sufficient to define category membership. For example, Lisker (1986) has reported that there are as many as 16 acoustic cues, all of which can be used to distinguish voiced vs. voiceless consonants (e.g., /ba/ vs. /pa/). Therefore, listeners must integrate multiple acoustic cues for speech categorization (Liberman et al., 1967; Liberman, 1996). Furthermore, there is high variability in these acoustic cues originating from different speech contexts, speaker's characteristics, among other sources. Adding to this complexity, temporal transitions of these acoustic cues occur at a millisecond scale that requires rapid tracking of simultaneous acoustic dimensions. These characteristics of the speech signal make it difficult to acquire explicit knowledge about the crucial acoustic dimensions that define speech categories. Therefore, learning of speech categories essentially represents learning of procedural knowledge that cannot be explicitly verbalized.

Since speech perception and learning inherently require integration of multiple, highly varying acoustic dimensions, explicit attempts to discover and integrate acoustic cues that are diagnostic to speech category identity may be extremely difficult. Yet, it has been shown that directed categorization training is likely to engage explicit/directed attention to acoustic features (Logan et al., 1991), and to recruit a sector of the basal ganglia (the head of the caudate nucleus) implicated in executive control and the cognitive processing of feedback (Tricomi et al., 2006). Learners are aware of the relationship between the outcome and speech categories in directed categorization training. Thus, they may attempt to discover potential features that may be critical for categorization in a declarative manner, which might not be optimal for learning speech categories due to their complex, difficult-to-verbalize nature (see Box 1A).

Box 1 **Feedback-based “Reward-Prediction Error” Learning.**

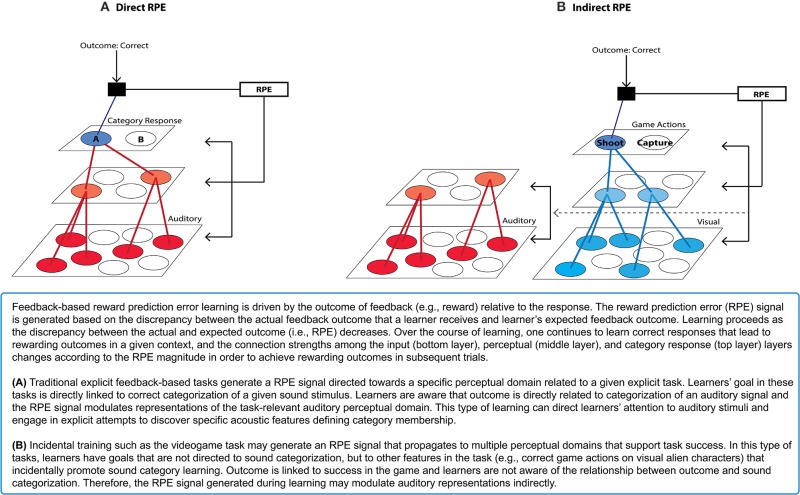

Within the domain of visual categorization, Ashby and colleagues have suggested that learning verbal rules (i.e., declarative knowledge) vs. integration of dimensions (i.e., procedural knowledge) that define categories is achieved by distinct, competitive learning systems (Ashby et al., 1998; Ashby and Ell, 2001; Ashby and Maddox, 2005). Learning declarative knowledge about the category features that are verbalizable engages executive attention and working memory, mediated by the prefrontal cortex and the anterior portion of the dorsal striatum (i.e., the head of the caudate nucleus). In contrast, acquisition of novel visual categories that require integration of multiple stimulus dimensions at some pre-decisional stage, referred to as “information-integration” categories, recruits posterior portions of striatum (i.e., the body and tail of caudate nucleus) that directly associate stimulus and response (e.g., Ashby et al., 1998; Ashby and Waldron, 1999; Ashby and Maddox, 2005). Because information-integration category input structures are designed so that no single dimension can independently signal the correct category membership, conscious effort to verbalize or explicit attempts to reason about the categorization decision are unhelpful, or even detrimental, to category learning (Ashby and Gott, 1988). Therefore, acquisition of information-integration categories becomes proceduralized instead of becoming reliant on working memory systems for explicit hypothesis-testing and allocation of executive attention to certain dimensions. This occurs via the posterior striatum such that direct associations between stimulus and response actions, implicitly acquired over the course of learning, are represented (Ashby et al., 1998; Yamamoto et al., 2013).

Both behavioral and neuroimaging findings have demonstrated that learning of information-integration categories recruits the direct stimulus-response association system associated with the posterior striatum to a greater extent than the explicit hypothesis-testing systems mediated by anterior striatum and the prefrontal cortex. In a behavioral study, Ashby et al. (2003) have found that switching stimulus-response key mappings in the course of training affected information-integration category learning, whereas explicit hypothesis-dependent category learning was unaffected. Similarly, compared to learning through variable response-category training (e.g., respond “yes” or “no” to “Is this A?” or “Is this B?”), consistent response mapping to stimulus category training (e.g., respond “A” or “B” to “Is this A or B?”) was more advantageous for information-integration category learning (Maddox et al., 2004). In addition, manipulations known to recruit explicit attention/working memory systems, such as variations in the amount of information or the temporal delay in the feedback, hamper learning of information-integration categories (e.g., Maddox et al., 2003, 2008). Functional neuroimaging studies have also found that information-integration visual category learning induces activation in the posterior striatum as well as in lateral occipital and inferior temporal areas to a greater extent than explicit-verbal category learning (Seger and Cincotta, 2005). More specifically, Nomura et al. (2007) have observed learning-related activity in the body of the caudate nucleus for learning visual information-integration categories. These studies provide direct evidence that learning of visual categories requiring integration of multiple dimensions is mediated by a qualitatively different system than learning declarative, explicit knowledge that directs attention toward specific stimulus features. This may further suggest that optimal learning of procedural knowledge about categories may be achieved by learning of direct stimulus-response associations via recruitment of the posterior portion of the striatum.

Learning visual information-integration categories has close resemblance to the acquisition of speech sound categories (Chandrasekaran et al., 2014) due to the highly multi-dimensional nature of speech categories. This suggests that training paradigms that model aspects of the natural environment, and which do not involve explicit speech sound categorization judgments and that discourage active attempts to reason about the category mappings, may be more effective than directed speech categorization training. Evidence supporting this point of view comes from several studies that have examined incidental auditory and speech category learning in the context of a videogame training paradigm (Wade and Holt, 2005; Leech et al., 2009; Lim and Holt, 2011; Liu and Holt, 2011) (Box 2). Unlike explicit feedback-based categorization tasks, the videogame task incorporates a number of characteristics that mimic, and perhaps amplify, relationships among advantageous cues available in natural learning environments. Participants encounter rich correlations of multimodal cues (i.e., consistent auditory-category to visual-object pairing) while navigating a virtual space-themed gaming environment. The game encourages functional use of sound categories because the categories signal which alien creature is approaching and thereby reveal the appropriate action to take. Feedback arrives in the form of success or failure in executing these actions (capturing or shooting the aliens), rather than explicit feedback about the correctness of an overt categorization response. Even without overt categorization of sounds or directed attention to the sounds, listeners exhibit robust learning of multidimensional, artificial nonspeech sound categories (Wade and Holt, 2005). Furthermore, the videogame training with these nonspeech sounds induces learning-related neural changes that mimic those observed in speech categories learning (Leech et al., 2009; Liu and Holt, 2011). This method of auditory categorization training is also effective for non-native speech category learning. Just 2.5 h of game training with non-native speech sounds evokes non-native speech category learning comparable to traditional laboratory training involving overt categorization and explicit feedback across 2–4 weeks (Lim and Holt, 2011). These findings suggest that aspects of the videogame task may effectively engage learning mechanisms useful for acquiring sound categories.

Box 2 **Videogame Training Paradigm (Wade and Holt, 2005).**

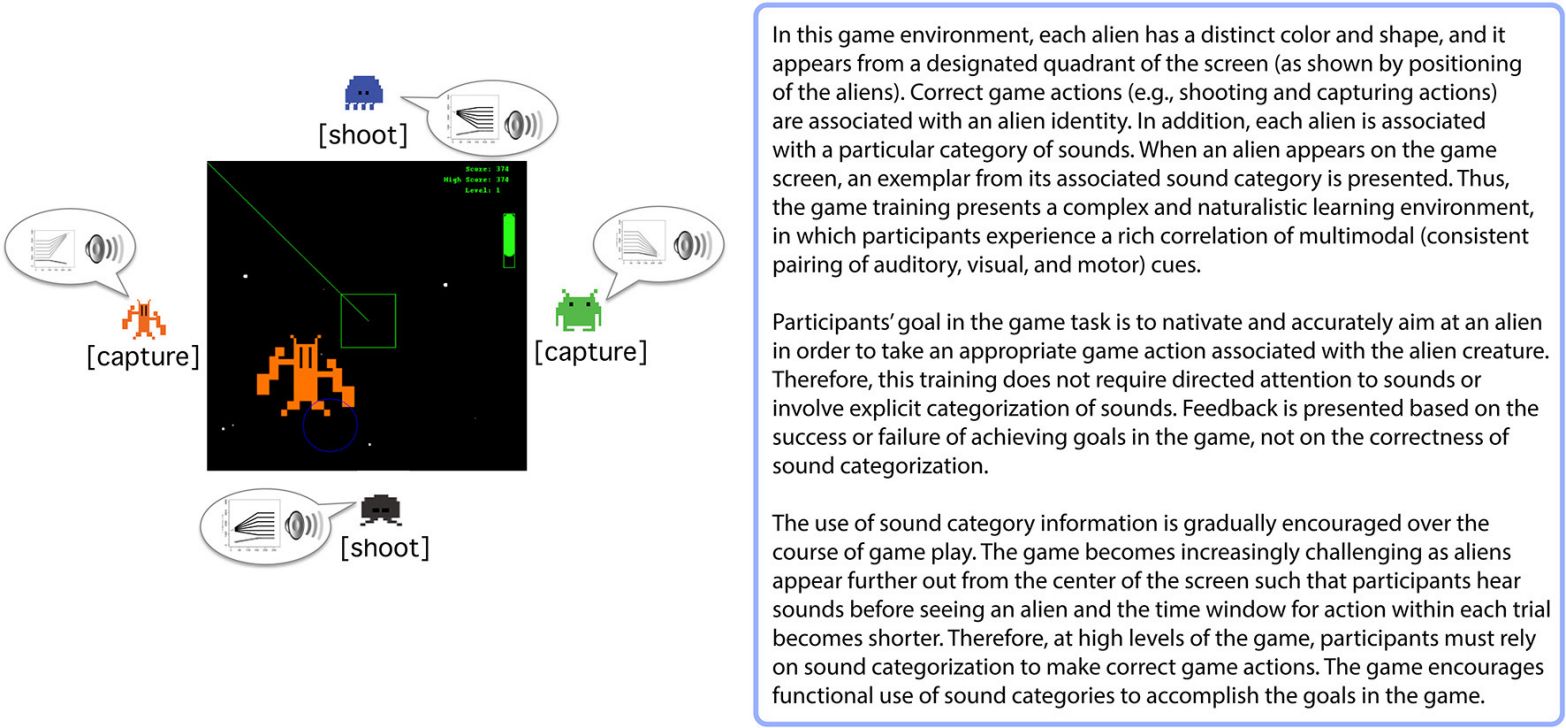

A significant element of this training may be participants' motivation to successfully navigate the videogame and execute capturing and shooting actions. Since these actions are not directed at sound categorization *per se*, the videogame training paradigm may elicit internally-generated reward prediction error feedback signals from the basal ganglia that indirectly induce changes in sound category representations that correlate to the success in the task (Box 1B). Processing task-relevant rewards incidentally in relation to sound categories may inhibit explicit attention to sounds, which can actually discourage perceptual learning (Tsushima et al., 2008; Gutnisky et al., 2009). Moreover, the increased engagement imposed by the game task requires faster execution of navigation and action responses. This task demand may distract individuals from making explicit hypotheses about specific acoustic features related to category mapping and, in turn, motivate learning automatic responses. Therefore, the Wade and Holt (2005) videogame may provide a training environment better-suited to recruiting the posterior striatal system that has been implicated in the learning of information-integration categories, as compared to directed categorization tasks. Supporting this possibility, we have found sound category learning within the videogame paradigm engages the posterior striatum (i.e., the caudate body) (Lim et al., 2013), which may contribute to learning-related perceptual plasticity (see Tricomi et al., 2006, discussion). This may explain the relative effectiveness of non-native speech category learning observed in the videogame (Lim and Holt, 2011), as compared to directed speech categorization training. These findings suggest that the basal ganglia play a role in learning within the Wade and Holt videogame task, and that its recruitment might be significant in supporting changes in cortical representations of the to-be-learned sound categories.

Another recent speech category learning study has emphasized the crucial role of reward-driven striatal-learning systems in non-native speech category learning. This study directly applied findings from the visual category learning literature (see Ashby and Maddox, 2005, for a review), which supports the existence of differential striatal learning systems recruited via principled manipulations to task structure and stimulus input distributions. By manipulating the schedule and content of trial-by-trial feedback, Chandrasekaran et al. (2014) have found that the extent of non-native speech category learning is greater in training tasks that tap into striatum-dependent procedural learning as compared to explicit hypothesis-testing learning. More specifically, compared to delayed feedback, immediate feedback occurring within 500 ms after a response can induce learning. This is hypothesized to occur because the 500-ms window aligns with the timecourse of influence of dopamine signals from feedback. Within this window, a brief dopamine signal can effectively influence cortico-striatal synapses for processing a stimulus and response while they remain active, which may enable learning of direct stimulus-response associations (see Ashby et al., 2007, for a review). Likewise, minimal information in the feedback (e.g., correct vs. incorrect) without information about the correct category mapping may minimize the chance of recruitment of the explicit hypothesis-testing process, and lead to greater engagement of the striatum-dependent procedural learning. Like the Wade and Holt (2005) videogame, this study also demonstrates that the nature of the task (in Chandrasekaran et al., 2014 the timing of feedback presentation) may modulate the recruitment of striatum-mediated learning, which can subsequently affect the outcome of non-native speech category learning.

Similarly, another line of research has demonstrated the effectiveness of implicit over explicit training procedures for perceptual learning. In studies of visual perceptual learning, some investigations have emphasized the role of diffuse reinforcement signals (specifically, dopaminergic reinforcement signals) in inducing perceptual plasticity and learning regardless of the direct relevance to the perceptual stimuli used in the task (Seitz and Watanabe, 2003, 2005, 2009; Seitz et al., 2009). Directly applying this paradigm, Vlahou et al. (2012) has shown that implicit, reward-contingent exposure of to-be-learned non-native speech stimuli seems to be more advantageous than explicit feedback-based exposure. Although this line of work has not implicated the striatum in learning, it has demonstrated the advantage of reward signals and of implicit vs. explicit training tasks for learning speech.

Overall, these results suggest that understanding the task demands and stimulus characteristics that effectively recruit the basal ganglia learning system can reveal approaches to promoting adult speech category learning. Regardless of whether the training paradigm involves overt, experimenter-provided feedback as in directed categorization tasks or indirect feedback as in the videogame task, the basal ganglia play a role in promoting learning based on outcome feedback. Significantly, however, differences in task characteristics may have important consequences for the manner by which learning is achieved (Box 1) inasmuch as they engage distinct basal ganglia-thalamo-cortical loops. Overt, category learning tasks that provide feedback about the accuracy of a speech category judgment may promote learning by directing explicit attention to sounds to discover critical stimulus characteristics relevant to category membership (Logan et al., 1991; Francis and Nusbaum, 2002; Heald and Nusbaum, 2014). Learning of explicit goal-directed actions based on feedback appears to be mediated by the anterior portion of the dorsal striatum, which interacts with executive and attention/working memory systems.

On the contrary, training tasks that recruit the posterior striatum may be advantageous for promoting optimal non-native speech category learning, because they may bypass an explicit hypothesis-testing system involving the anterior striatum, and instead promote a form of procedural learning that is more suited for learning categories with an information-integration structure, including speech categories (Chandrasekaran et al., 2014). One possible advantage of posterior striatum recruitment in category learning is that it can interact with sensory cortex to a greater extent than the anterior striatum, for which interaction with sensory cortex is mediated through the frontal cortex. Learning of implicit stimulus-action relationships appears to involve striatal regions in the posterior striatum, which are known to develop automatic responses based on consistent reward experiences (Seger and Cincotta, 2005; Cincotta and Seger, 2007; Kim and Hikosaka, 2013; Yamamoto et al., 2013), thereby prohibiting the use of non-optimal strategies for categorization. Therefore, the Wade and Holt (2005) videogame task may indirectly promote learning of sound category features even as listeners' attention is directed away from the sounds and toward other task goals, such as making correct game actions to respond to the visual aliens. The task demands of the primary task (navigating the videogame, for example) may be time and resource demanding enough to discourage active attempts to reason about category-diagnostic dimensions. Or, learners might be truly unaware that the outcomes of their actions are linked to the learning of category-relevant features. Future investigations are needed to clarify the role of the posterior striatum in category learning, specifically regarding the mechanisms by which category learning is actually achieved and the nature of learned categories represented in the posterior striatum.

## Basal ganglia interactions with sensory cortex

Previous neuroimaging studies involving auditory category learning have shown that category learning can change cortical processing for the learned sounds. In particular, the observed effect of feedback valence on the activation of the auditory regions in the superior temporal gyrus (Tricomi et al., 2006) may suggest that processing of feedback information via the basal ganglia can induce changes in the sensory cortical regions for learned phonetic representations. For example, incidental learning of nonspeech sound categories within the Wade and Holt (2005) videogame recruits posterior superior temporal sulcus (pSTS) regions associated with speech processing in response to the newly-acquired nonspeech categories (Leech et al., 2009). This change may be occurring at an early processing stage, as the same category learning can elicit changes in the evoked response potential within 100-ms after the onset of the learned sounds (Liu and Holt, 2011). Furthermore, explicit feedback-based training of sound categories has been shown to promote activity changes in the auditory cortical regions, such that they respond in a categorical fashion (e.g., Callan et al., 2003; Golestani and Zatorre, 2004; Dehaene-Lambertz et al., 2005; Desai et al., 2008; Liebenthal et al., 2010; Lee et al., 2012; Ley et al., 2012). The observed learning-related changes of sensory cortical processing suggests that the sensory cortex is affected by “teaching signals” elicited from training (e.g., reward-based learning signals based on feedback). The basal ganglia may support such interaction with the sensory regions.

As noted earlier, the basal ganglia are known to have multiple anatomical cortico-striatal loops that innervate widespread areas of the cerebral cortex, including motor, cognitive and perceptual regions (see Alexander et al., 1986, for a review). These loops are organized in a topographical manner such that information in each loop projects to specific regions in the striatum and in the thalamus. This information is subsequently fed back to distinct cortical regions (Parent and Hazrati, 1995) via “closed loops,” which send reciprocal projections to the originating cortical regions (Alexander et al., 1986) and “open loops,” which ultimately terminate at different cortical regions (Joel and Weiner, 1994). These anatomical loops serve distinct functions, the nature of which depends on the pattern of cortical projections. Among these multiple cortico-striatal loops, the visual loop from inferior temporal regions of cerebral cortex has been commonly implicated in perceptual category learning (see Seger, 2013, for a review; Figure 2). Although auditory regions in the superior temporal region form cortico-striatal projections similar to the visual loop, the auditory loop has been relatively less studied. Therefore, we first focus on the findings from the visual cortico-striatal loop, which would be relevant for understanding the role of the auditory cortico-striatal loop inasmuch as they reveal how posterior sites of basal ganglia may influence sensory cortical processing.

The presence of the visual cortico-striatal loop indicates that the striatum is able to interact with cortical regions responsible for sensory processing. Animal neurophysiology studies have demonstrated that the body and tail of the caudate nucleus contain neurons that respond to visual input. Studies examining the function of this visual loop have shown that animals with specific lesions in the tail of the caudate are impaired in visual discrimination learning (Packard et al., 1989; Packard and McGaugh, 1992). Another study has shown that among all connections from the visual cortex, only connections between the inferior temporal cortex and the striatum are necessary and sufficient to achieve visual discrimination learning (Gaffan and Eacott, 1995).

Human neuropsychological and neuroimaging studies have provided converging evidence to support the role of the striatum in visual category learning. Studies have shown that Parkinson's and Huntington's disease patients are impaired in learning visual categories that require information integration (Filoteo et al., 2001; Ashby and Maddox, 2005). Human fMRI studies have demonstrated recruitment of the body and tail of caudate nucleus during visual categorization (Cincotta and Seger, 2007; Nomura et al., 2007). These converging findings from both animal and human research demonstrate the role of the striatum (specifically, the body and tail of the caudate nucleus) in category learning within the domain of visual perception. Based on the fact that reward-related learning within the striatum can modulate synaptic efficacy across relevant cortico-striatal loops (Houk and Wise, 1995), the striatum might play a significant role in inducing learning-related representational changes in visual cortex.

It is of note that striatal-mediated visual category learning research has mostly focused on “open loop” projections of cortico-striatal pathways. Research typically has assumed that perceptual representations are computed and selected by the visual cortex whereas the striatum is responsible for selecting an appropriate category decision, which is then transmitted to motor cortex to execute a response (Ashby et al., 1998; Ashby and Waldron, 1999; Ashby and Spiering, 2004). In other words, most research has been directed at how basal ganglia-dependent circuits acquire information that can be used to guide “action selection” in response to a visual stimulus (see Seger, 2008, for a review). Therefore, these studies have often been concerned with interactions among different cortico-striatal loops: projections from the sensory regions (i.e., high-level visual regions) to the striatum, and projections from the striatum to frontal or motor cortical regions (Lopez-Paniagua and Seger, 2011). In contrast, relatively less attention has been directed to the role of the “closed” striatal projection back to visual cortex (or sensory cortex, in general). An animal viral tracing study has shown that the basal ganglia system indeed projects back to the inferior temporal cortex (Middleton and Strick, 1996), the high-level visual cortical region that plays a critical role in visual recognition and discrimination (Mishkin, 1982; Ungerleider and Mishkin, 1982) and visuomotor associations (Mishkin et al., 1984). In humans, damage to the visual loop striatal circuitry has been associated with deficits in face perception (Jacobs et al., 1995). This evidence indicates that the striatum has the capacity to influence sensory processing within visual cortex.

The striatum may affect visual processing through dopamine-dependent synaptic plasticity within the basal ganglia (Kerr and Wickens, 2001; Centonze et al., 2003; Calabresi et al., 2007). A neurocomputational model proposed by Silkis (2007, 2008) shows that reorganization of the synaptic network via dopamine can differentially modulate the efficiency of strong and weak cortico-striatal inputs in a manner analogous to the basal ganglia's role in action selection. When strong visual cortico-striatal input occurs simultaneously with dopamine release, the basal ganglia circuit can be reorganized to ultimately disinhibit the visual cortical neurons that were strongly activated, and conversely inhibit neurons that were weakly activated. Therefore, if either top-down or bottom-up visual attention can evoke dopamine release (Kähkönen et al., 2001), the cortico-basal ganglia network may be reorganized to affect processing that occurs within visual regions. Through this type of mechanism, feedback-based dopaminergic reinforcement signals from the training experience could affect sensory processing regions via the basal ganglia. In support of this argument, dopamine release associated with the receipt of reward can affect early sensory/perceptual processing. Incidental delivery of reward during passive viewing of visual stimuli has been shown to induce changes in low-level visual discrimination. Perceptual sensitivity is selectively increased to process features of a stimulus that were simultaneously presented with reward, whereas there was no change in sensitivity to process unrewarded stimuli features (Seitz and Watanabe, 2003, 2009; Seitz et al., 2009).

Another possible mechanism by which the striatum could interact with sensory cortex is via the prefrontal cortex. As noted in section Overview of the Basal Ganglia and Reinforcement Learning, the basal ganglia effectively learn stimulus-action-outcome associations leading to rewards via dopamine release. This reward-related stimulus-action representation may reside in frontal higher-order cognitive or motor regions. Across various learning studies, the prefrontal cortex is known to represent “goal-directed” actions in response to a given stimulus (Petrides, 1985; Wallis et al., 2001; Muhammad et al., 2006). It has been proposed that this learning in the prefrontal cortex is achieved through recurrent interaction with the basal ganglia; reward-driven stimulus-response associations rapidly acquired by the basal ganglia are projected to the prefrontal cortex through a cortico-striatal loop, while the prefrontal cortex slowly integrates and binds multiple information sources to build higher-order representations (i.e., the process of generalization) (Pasupathy and Miller, 2005; Miller and Buschman, 2008). Therefore, in the context of category learning, the basal ganglia may induce a “goal-directed” representation of appropriate category response toward a given stimulus in the prefrontal cortex (Kim and Shadlen, 1999; Freedman et al., 2001; McNamee et al., 2013), which in turn may exert top-down attentional modulation on sensory regions to selectively respond to learning-relevant sensory information (Duncan et al., 1997; Desimone, 1998). It remains unclear whether the frontal cortex exerts a direct influence on the sensory regions or whether top-down attention modulates plasticity of the cortico-basal ganglia-thalamic circuit via dopamine release (see Miller et al., 2011, discussion; Skinner and Yingling, 1976; Silkis, 2007). Either possibility invites consideration of the role of the basal ganglia in indirectly or directly modulating attention (van Schouwenburg et al., 2010), which can ultimately tune sensory cortex to form robust category representations (Fuster et al., 1985; Beck and Kastner, 2009) and to exhibit experience- and learning-dependent neural response selectivity to category-relevant over category-irrelevant sensory features (e.g., Sigala and Logothetis, 2002; Op de Beeck et al., 2006; Folstein et al., 2013; van der Linden et al., 2014).

These loops provide a means by which the striatum can interact with sensory cortical regions and may indicate a role for the basal ganglia in auditory/speech category learning. Compared to the role of visual cortico-striatal loop, relatively less is known about auditory cortico-striatal loop that links auditory cortical regions and the basal ganglia. Nevertheless, animal neurophysiological research has shown a direct link between the striatum and auditory cortex, which strongly implies the presence of an auditory cortico-striatal loop. Within the body of the caudate, auditory cortex projections converge onto a region that is distinct from the striatal site receiving cortical projections from visual processing regions (Arnauld et al., 1996). The sector of the striatum that receives auditory cortical projections projects back to the auditory cortex via the output structures of the basal ganglia (Parent et al., 1981; Moriizumi et al., 1988; Moriizumi and Hattori, 1992; see Parent and Hazrati, 1995, for a review). Non-human primate neurophysiology studies also have demonstrated that different auditory cortex regions (i.e., primary, secondary) form connections with different sectors of the striatum (Van Hoesen et al., 1981; Yeterian and Pandya, 1998). Importantly, a recent study has demonstrated in rats that auditory cortico-striatal projections influence behavioral performance during a reward-based frequency discrimination task (Znamenskiy and Zador, 2013).

There is also emerging evidence from human neuroimaging revealing the role of the auditory cortico-striatal loop. Geiser et al. (2012) have shown that recruitment of a cortico-striatal system facilitates auditory perceptual processing in auditory temporal cortex. Directly relevant in the context of learning speech categories, Tricomi et al. (2006) observed that observed recruitment of the striatum among native Japanese adults learning of English /r/ and /l/ categories via an overt categorization task with feedback. This study demonstrated a possible interaction between striatum system and the auditory cortex, such that differential activity was observed in the caudate nucleus as well as in the left superior temporal gyrus, a cortical region known to be associated with non-native phonetic learning (Callan et al., 2003; Golestani and Zatorre, 2004), across correct vs. incorrect trials. Although it is still unclear whether the recruitment of the striatum in the overt categorization task involves the top-down influence from the higher-order cortical regions (e.g., frontal cortex) or a direct influence from the striatum to auditory regions, this evidence may indicate that the striatum, recruited by feedback-based training tasks, interacts with cortical regions processing speech. This striatal innervation in learning may effectively induce learning-related plasticity, which may ultimately influence cortical representations of the newly learned non-native speech categories.

In addition to the striatal interaction with the auditory processing regions via the “closed” auditory loop, the “open loop” pathway of the basal ganglia to frontal and motor regions may contribute to speech category learning by facilitating sensory and motor interactions. Previous neuroimaging studies investigating speech perception have demonstrated interactions between the speech perception and production (i.e., sensory and motor interactions). For example, listening to speech sounds activates both auditory regions (i.e., superior temporal cortex) and motor regions involved in speech production (e.g., Wilson et al., 2004; Wilson and Iacoboni, 2006). Perception of distinct speech categories is reflected in neural activity patterns in the frontal and motor regions including Broca's area and pre-supplmentary motor area (pre-SMA), known to participate in speech motor planning and articulatory processing (Lee et al., 2012). Moreover, learning non-native speech categories has also been shown to engage similar regions in the frontal and motor areas (Callan et al., 2003; Golestani and Zatorre, 2004), which interact with the basal ganglia via cortico-striatal loops (Alexander et al., 1986; Middleton and Strick, 2000; Clower et al., 2005). Although the nature of the speech perception and production link (see Lotto et al., 2009, for a review) and its role in speech category acquisition are yet to be discovered, the basal ganglia's closed and open loop projections have the potential to facilitate learning of speech categories via interactions between perception- and action-related representations of speech categories.

## Category generalization through convergence of the basal ganglia

Previous studies investigating basal ganglia-mediated category learning have emphasized the learning of representations at the level of category decision-making to trained exemplars (e.g., Ashby et al., 1998). Therefore, it remains uncertain whether the basal ganglia contribute to forming perceptual category representations that are generalizable across variable instances of a class (Palmeri and Gauthier, 2004). This is an important issue for speech category learning, as generalization of learning to new exemplars is a hallmark of categorization. Although there might be multiple factors that can contribute to generalization (e.g., attentional modulation), the basal ganglia may play a crucial role.

Cortical information funnels through the basal ganglia via multiple cortico-striatal loops. Massive projections from widespread cortical areas are reduced as they reach the striatum and globus pallidus. The number of neurons from cortex to the striatum is reduced on the order of 10 (Zheng and Wilson, 2002), which is further reduced at the globus pallidus on the order of 10^2^–10^3^ (Percheron et al., 1994), thereby creating a highly convergent “funneling” of information within the basal ganglia (Flaherty and Graybiel, 1994). With this convergence of cortical input to the basal ganglia approximately at a ratio of 10,000:1 (Wilson, 1995), compressed cortical information is fed back to the cortical regions that send projections to the striatum via basal ganglia output.

The exact degree and the pattern of this convergence have been under debate. Initially, the cortex was thought to innervate the striatum in a topographical fashion such that a group of spatially adjacent cortical input would project to a localized region within the striatum (Webster, 1961), thus removing redundancy of the input. However, the later findings have shown that the striatum is innervated by distributed, yet inhomogeneous, cortical input (Selemon and Goldman-Rakic, 1985; Malachi and Graybiel, 1986), whereby the striatum acts as a “pattern detector” across cortical input (Zheng and Wilson, 2002; Bar-Gad et al., 2003). In other words, a specific pattern of cortical input even originating from spatially sparse cortical regions may be required to activate corresponding striatal neurons. In this way, the striatum may represent functional organization, rather than the spatial topography of the cortex (e.g., Flaherty and Graybiel, 1993, 1994). Although such a pattern of innervation can raise questions about the extent of convergence, the compression of cortical information within the striatum is inevitable. With the reduced number of striatal neurons, the striatum cannot represent all possible patterns of cortical input (Zheng and Wilson, 2002). This constraint allows the basal ganglia to reduce or compress cortical information, which is eventually fed back to the cortex.

This converging characteristic of the basal ganglia might be quite suitable for generalization by preserving learning-relevant information and diminishing stimulus-specific information. The computational model by Bar-Gad et al. (2003) illustrates this dimension reduction mechanism of the basal ganglia; as information is reduced, reward-related information is retained and enhanced whereas non-rewarded information is inhibited or unencoded. This computational scheme could be useful for forming category representations capable of producing generalization across variable instances by strengthening category-relevant over -irrelevant information within sensory cortex, via recurrent projections with the basal ganglia.

The basal ganglia's potential role in information reduction could provide a useful and important neural mechanism for the facilitation of perceptual category learning. Across visual and auditory domains, perceptual category learning studies have emphasized the importance of stimulus variability in acquiring robust and “generalizable” category formation. Posner and Keele (1968) have observed that training with high-variability stimuli during visual pattern classification task is more advantageous than training with low-variability stimuli, as assessed by the ability to generalize learning to accurately classify novel visual patterns. Similarly in the domain of speech category learning, studies have emphasized the benefits of high-variability in training stimuli (with speech from multiple talkers, and speech contexts, e.g., Logan et al., 1991; Lively et al., 1993, 1994) as training with low-variability fails to generalize listeners' learning to novel sounds. There is a perceptual cost associated with learning categories from multi-speaker stimuli as it can lead to increased response times and reduced overall categorization accuracy (Mullennix et al., 1989). Nevertheless, training with low-variability (e.g., single-speaker's speech) stimuli may lead to non-optimal category learning dependent on information diagnostic to that speaker's speech, while training with multi-speaker stimuli can highlight category-relevant acoustic cues. Because highly variable stimulus input can create enough variance in category-irrelevant dimensions, learners may selectively encode less-variable, but category-relevant dimensions to form representations that effectively capture the information most diagnostic of category membership (Lively et al., 1993; see Pisoni, 1992), which can be applied upon encountering novel instances. The mechanism of high-variability training promoting perceptual category learning has a close resemblance to the basal ganglia's potential role in input dimension-reduction.

The dimension reduction characteristic of the basal ganglia may serve a beneficial role in natural speech category learning. A main challenge of speech perception/categorization is parsing highly variable acoustic signals as linguistically-relevant units (see Holt and Lotto, 2010, for a review). As mentioned above, speech is inherently multidimensional such that many acoustic cues can be used to determine category membership. However, it is important to note that although multiple cues covary with speech category identity, not all acoustic cues are equally weighted for perception; listeners rely on certain acoustic dimensions more heavily than others for categorization (Francis et al., 2000; Idemaru et al., 2012). Based on the distributional characteristics of speech categories in a given language, listeners learn to rely more on acoustic dimensions that are most diagnostic of category membership. Of course, there might be an accumulation of experience with statistical regularity of the speech category input (i.e., similarity across exemplars within a category; see computational models by McMurray et al., 2009; Toscano and McMurray, 2010). Nevertheless, there appears to be a prioritizing of category-relevant dimensions in speech perception. The mechanism of information reduction via cortico-striatal convergence may serve a supportive role for facilitating extraction of critical and behaviorally significant information relevant for categorization. This mechanism may give rise to robust perceptual representations.

## General concerns and future directions

### Learning-related representations

It is of note that there exist discrepancies among independent lines of research in perceptual category learning and basal ganglia-mediated category learning research. General perceptual category and object learning studies have been concerned largely with observations of learning-related neural changes in the sensory cortices as an outcome of learning. Perception (and sensory cortex) is tuned to exhibit a selective improvement in processing category-relevant over -irrelevant dimensions (Goldstone, 1994; Gureckis and Goldstone, 2008). In contrast, basal ganglia-mediated category learning research has mostly been concerned with issues regarding how perceptual categories are acquired, with the presumption that learning-related representational change occurs at the level of action selection and decision making about a given category instance (i.e., associations between a stimulus and a correct categorization response), leaving sensory representations relatively unaffected (e.g., Ashby et al., 1998; Ashby and Waldron, 1999; Ashby and Spiering, 2004). Because of this orientation, previous studies have indicated the basal ganglia in category learning regardless of the presence of category structure. These studies have not differentiated or directly compared the process of learning structured categories that require integration of multiple dimensions vs. arbitrary/unstructured category exemplars randomly distributed without any specific category boundaries (Seger and Cincotta, 2005; Cincotta and Seger, 2007; Seger et al., 2010; Lopez-Paniagua and Seger, 2011; Crossley et al., 2012), although different category input distributions can have a notable impact on sensory processing and learning (Wade and Holt, 2005; Holt and Lotto, 2006; Lim et al., 2013).

A similar tension exists in interpreting results of perceptual category learning studies. Some studies have demonstrated neural changes in sensory regions after learning (e.g., Sigala and Logothetis, 2002; Guenther et al., 2004; Desai et al., 2008; Ley et al., 2012; van der Linden et al., 2014), even when listeners are passively exposed to learned category instances after training (Leech et al., 2009; Liu and Holt, 2011). On the contrary, instead of sensory regions, other studies have suggested that learned categories and objects are represented in the higher-order cortical areas like frontal regions (e.g., Freedman et al., 2001, 2003; Jiang et al., 2007). This view is in line with basal ganglia-mediated category learning research that posits that the learning-related representational change occurs only at the level of action selection and decision-making. As such, the target of category-learning representational change is as yet unknown. However, it is important to acknowledge that that learning-related plasticity arising either in sensory cortical processing or other decision-related cortical regions may depend critically on how perceptual categories are defined (Folstein et al., 2012) and the tasks by which they are learned.

Future research will be needed to resolve whether category learning is better conceived of as change in decision mapping vs. sensory perception and to determine whether both types of representational change may be simultaneously developed over the course of learning via multiple cortico-striatal loops. This possibility would lead to learned stimulus-response associations to strengthen the behavioral significance of perceptual representations, which perhaps could induce changes in the sensory-level processing to selectively enhance perception of category-diagnostic features.

### Naturalistic learning environments for speech

Although the basal ganglia have been implicated in visual category learning, their role has been rarely considered in understanding speech category learning. The discussion above highlights some reasons to believe that characteristics of basal ganglia function may support second-language speech category learning under the right task demands. An open question is whether this system might support first-language speech category learning. Infants fairly rapidly attune to the distributional regularities of native language speech categories without explicit instruction (e.g., Aslin et al., 1998; Maye et al., 2002). A common notion is thus that infants acquire native speech categories without feedback, perhaps through mechanisms related to statistical learning (see Kuhl, 2004, for a review). Since infants exhibit statistical learning in passive listening laboratory tasks (e.g., Saffran et al., 1996, 1999; Aslin et al., 1998; Maye et al., 2002), other learning mechanisms have not been widely considered.

However, an important concern is whether the learning systems engaged by passive laboratory tasks would scale up to accommodate the complexity of natural language learning environments. In a natural listening environment, listeners experience highly acoustically-variable phonemic sounds in fluent and continuous speech rather than as isolated instances. This adds the additional challenge of learning the perceptual mapping of sound to functionally equivalent language-specific units (such as phonemes, or words) while simultaneously parsing continuous speech input. In addition, speech exposure often occurs within complex visual scenes for which there are multiple potential referents, creating additional learning challenges (Medina et al., 2011). This complexity introduces an explosion of potentially-relevant statistical regularities, leading some to suggest that passive computation of statistics in the speech input alone cannot induce early speech learning within complex natural speech settings (Kuhl, 2007). Evidence suggests that statistical learning within natural language environments may be supported by modulation from attentional and motivational factors (Kuhl, 2003; Kuhl et al., 2003; Toro et al., 2005), contingent extrinsic reinforcers like social cues (Goldstein et al., 2003; Gros-Louis et al., 2006), and the presence of correlated multimodal (e.g., visual) inputs (Hollich et al., 2005; Teinonen et al., 2008; Yeung and Werker, 2009; Thiessen, 2010). Similar to the learning process engaged by the videogame training, the indirect influence of such signals on early speech processing may indicate a potential role for recruitment of the basal ganglia learning system that incidentally facilitates acquisition of native speech categories. Investigating this further in future research will help to refine models of first-language speech category acquisition.

A different line of research has suggested that implicit, task-irrelevant perceptual features of rewarded stimuli can be learned with passive exposure via a diffuse dopamine signal (Seitz and Watanabe, 2003, 2005; Seitz et al., 2010). Although this line of research has not implicated the specific role of the striatum, Vlahou et al. (2012) demonstrates the importance of reward-related learning signals on perceptual plasticity (Seitz et al., 2009) useful for non-native speech category learning. However, it is of note that the task-irrelevant training paradigm does not have any component to signal information about the functional distinctiveness across different categories or to induce reward or dopamine signals throughout learning, except for the external rewards that are implicitly paired with the stimuli by the experimenter. This task-irrelevant perceptual learning may lead to perceptual attunement to very specific stimulus information that coincides with external reward delivery. Due to such specificity, non-native speech learning in this task seems to be limited to familiar training speech sounds that have been paired with external rewards and does not generalize to novel sound stimuli (Vlahou et al., 2012). Although the thresholds of non-native speech sound discriminability change as a result of this training, it is not yet known whether task-irrelevant perceptual learning can lead to perceptual *category* learning and generalization. Nonetheless, although research on task-irrelevant perceptual learning does not yet converge with the learning challenges of non-native speech category learning, it does provide insight in the learning systems that may be engaged to modify sound perception. It may be fruitful to try to bridge this gap in future research.

The Wade and Holt (2005) videogame training paradigm described above also falls short in modeling the naturalistic learning environment for learning speech categories. However, it does provide a means of manipulating signals influential in first language speech category acquisition such as motivational factors, contingent reinforcement, and multimodal correlations. It also presents the possibility of scaling up the learning challenges. In recent research Lim et al. (under review) have found that adults can discover non-native speech and also nonspeech sound categories from continuous, fluent sound input in the context of the Wade and Holt (2005) videogame. This learning generalized to novel exemplars, indicative of robust category learning. Given that research implicates the basal ganglia in learning within this task (Lim et al., 2013), there is the opportunity for future research to compare and contrast basal ganglia-mediated learning with that arising from passive learning.

## Conclusion

The basal ganglia are a very complex and intricate neural structure, consisting of multiple sub-structures that interact with most cortical areas through diverse connections. The structure has been highly implicated in motor functions. However, general learning studies outside of the speech/auditory domain have revealed its contribution to cognitive functions, particularly in learning from external feedback to form goal-directed and procedural behaviors as well as learning visual categories.

In the domain of speech category learning and elsewhere, research commonly uses explicit feedback-based tasks to induce effective learning. Although this type of task engages the basal ganglia system during learning, and is known to be effective for acquisition of non-native speech categories (McCandliss et al., 2002; Tricomi et al., 2006), speech learning studies have put relatively less emphasis on the nature of the training experience influencing the learning process and outcome. Likewise, existing neurobiological and computational models of speech processing (e.g., the dual-stream neural account of Hickok and Poeppel, 2004; or the TRACE computational model of McClelland and Elman, 1986, but see Guenther, 1995) have focused on cortical networks and have not widely considered how subcortical structures like the basal ganglia participate in speech category acquisition or captured more than limited forms of learning. Although it has great relevance, current theories do not address the role of different training experiences on recruiting the basal ganglia and the corresponding effects on behavioral and neural changes for speech perception and learning. Therefore, a better understanding of learning-related functions of the basal ganglia system may be important in elucidating how effective speech category learning occurs. This may have rich benefits for optimizing training environments to promote perceptual plasticity in adulthood. Furthermore, understanding of the basal ganglia system may provide a broader understanding of language learning in general as it has been implicated in various aspects of language-related processing (Ullman et al., 1997; Doupe and Kuhl, 1999; Kotz et al., 2009).

The topics of speech perception and learning, and basal ganglia-mediated category learning, have been largely studied independently. Speech perception, once considered a “special” perceptual system, has only recently begun to be studied in a manner that fully incorporates general cognitive/perceptual learning research on the development of perceptual representations. On the other hand, studies of basal ganglia function with regard to category learning have emphasized understanding of the process of learning category-relevant decisions rather than learning-related changes in perceptual organization. However, these separate lines of research share commonalities. We have attempted to argue that there is great potential in bridging efforts to understand speech perception and learning with general cognitive neuroscience approaches and neurobiological models of learning.

### Conflict of interest statement

The authors declare that the research was conducted in the absence of any commercial or financial relationships that could be construed as a potential conflict of interest.
